# Care Received by Migrant Women Who Died During or After Pregnancy: A National Confidential Case Note Review

**DOI:** 10.1111/1471-0528.70288

**Published:** 2026-06-23

**Authors:** Kerrie Stevenson, Kemi Ogunlana, Nirupama Prabhakar, Brenda Kelly, Allison Felker, Fiona Stevenson, Robert W. Aldridge, Sara Kenyon, Marian Knight, Nicola Vousden

**Affiliations:** ^1^ Great Ormond Street Institute of Child Health University College London London England UK; ^2^ National Perinatal Epidemiology Unit, Nuffield Department of Population Health University of Oxford Oxford England UK; ^3^ The Oxford Rose Clinic Oxford University Hospitals NHS Foundation Trust Oxford England UK; ^4^ Primary Care and Population Health University College London London England UK; ^5^ Institute for Health Metrics and Evaluation University of Washington Seattle Washington USA; ^6^ Institute for Health Informatics University College London London England UK; ^7^ School of Health Sciences University of Birmingham Birmingham England UK

**Keywords:** inequities, maternal health, migration

## Abstract

**Objective:**

To co‐produce a bespoke maternity care checklist for recently arrived migrant women in the UK. To use this to describe the maternity care received by recently arrived migrant women who died during or up to six weeks after pregnancy over the past decade in the UK, and generate hypotheses about areas for improvement.

**Design:**

Confidential national review of medical records.

**Population:**

Thirty‐nine recently arrived pregnant or postnatal migrant women died in the UK between 2012 and 2021.

**Methods:**

Maternal deaths amongst recently arrived migrant women were identified using UK Confidential Enquiry into Maternal Deaths and Morbidity (MBRRACE‐UK) data. A national case note review was conducted using a co‐produced migrant maternity care checklist developed with input from service users.

**Main Outcome Measures:**

Assessment against the migrant maternity care checklist.

**Results:**

Amongst those eligible for antenatal care, most (70%, *n* = 19/27) attended their first appointment after 13 weeks gestation. Amongst women needing an interpreter, 42% (*n* = 8/19) had professional interpreting services during antenatal care, 19% (*n* = 3/16) in intrapartum care, and 0% (*n* = 0/9) in postpartum care. Two fifths of women had documentation suggestive of discrimination or racism (41%, *n* = 16/39). Potential areas for improvement included access to formal interpreting services; access to first antenatal appointments before 13 weeks; and follow‐up after non‐attendance.

**Conclusions:**

Results should be interpreted with caution given the small number of women included and the descriptive and hypothesis generating nature of this paper. However, this study identifies areas of care that are important to women and professionals and could be prioritised for further research and improved practice.

## 1 Introduction

In 2023, 15% of the UK's total population was born outside the UK [1, 2]. In the same year, 32% of women giving birth in England and Wales were not born in the UK [3]. Despite this, we have limited population‐based maternity outcome data for these women. At present, the NHS does not record migration status in records, so most population‐based analyses rely on proxy measures of migration status [4]. Globally, studies suggest some migrant women and their children, particularly forced migrants, experience adverse increased morbidity and mortality compared to host country populations [5, 6, 7, 8, 9, 10, 11]. Reasons for this include dangerous migration journeys, exposure to trauma, experiencing racism, language barriers, and poverty [12, 13, 14, 15, 16].

Within high‐income countries, systematic review data suggest that forced migrant women are more likely to present later in pregnancy and miss antenatal appointments compared to the host country population [5]. They are also at higher risk of miscarriage and stillbirth, and their babies experience higher perinatal mortality [5]. Although no population‐based data exist in the UK, small scale studies and qualitative research have highlighted significant disparities in maternity care for forced migrant women compared to UK‐born women, including experiencing barriers to accessing interpreters, harm arising from reliance on family members for interpretation, charges for NHS maternity care impacting trust in services, and issues navigating the National Health Service (NHS) [17, 18, 19, 20, 21, 22].

The UK Confidential Enquiry into Maternal Deaths and Morbidity delivered by the MBRRACE‐UK collaboration includes national surveillance of maternal mortality and morbidity. Similar to previous years, recent results indicated that approximately 30% of the women who died in 2020–22 were born outside of the UK, but little is known about their maternity experiences or outcomes [23]. Included within the 2020–22 MBRRACE‐UK report, was a confidential enquiry into the care of recently arrived migrant women who experienced a morbidity but did not die [21]. Key findings were that recently arrived migrant women had limited access to interpreters, presented late for antenatal care, and were not followed up after non‐attendance. However, prior to this current study, there has been no exploration of the characteristics of the recently arrived migrant women who died in the UK or a formal assessment of the care received. This study aimed to:

1. Describe the demographics and past medical and obstetric histories of recently arrived migrant women who died during or up to six weeks after pregnancy over the past decade in the UK.
2. Co‐create a bespoke maternity care checklist for recently arrived migrant women.
3. Use this checklist to describe care received by this cohort of recently arrived migrant women who died in the UK over the past decade and generate hypotheses on how to improve maternity care for this population.

## 2 Methods

### 2.1 Patient Involvement and Inclusivity

Our authorship teams include migrant mothers with lived experience of UK maternity care. These women helped conceptualise and interpret these data to maximise contextual insight. The terms ‘pregnant women’ and ‘mothers’ will be used throughout this review to reflect the recorded characteristics of individuals identified in the medical records, but the authors recognise that not everyone who is pregnant or giving birth will identify as a woman or a mother.

### 2.2 Data Sources

In this confidential review of medical records, any maternal death amongst a recently arrived migrant woman (less than two years in the UK before their death) during or up to six weeks post‐pregnancy in the UK from 01/01/2012 to 31/12/2021 was eligible for inclusion. Maternal deaths are identified through a variety of sources [24]. The majority are notified to the MBRRACE‐UK office directly from the hospital where the mother died, and others are notified from a variety of sources such as Coroners/Procurators Fiscal, pathologists, and members of the public. Data held by MBRRACE‐UK are cross‐checked against national birth and death registries to ensure all maternal deaths are ascertained. Full anonymised medical records obtained from the hospital where the mother died and MBRRACE‐UK surveillance data were analysed.

### 2.3 Descriptive Analysis

Demographic characteristics were obtained from routine MBRRACE‐UK surveillance data and supplemented with information extracted from women's medical records. Decisions on which characteristics to include were based on previous literature and engagement with service users and clinical, non‐governmental, and policy stakeholders. Income categories of countries of origin were assigned using the World Bank Income Groupings [25].

### 2.4 Development of the Migrant Maternity Care Checklist

The case note review used a newly developed checklist of high quality maternity care for recently arrived migrant women. This checklist was co‐created with service users, midwives, obstetricians, researchers, and policymakers through a five‐stage iterative process, incorporating published literature and clinical guidelines. Initially, two focus groups engaged 25 migrant women with lived experience of UK pregnancy care, selected via purposive sampling to ensure diversity in migration status, country of origin, and language. Women self‐defined high quality care for recently arrived migrant women by reflecting on their desired antenatal, peripartum, and postnatal support alongside clinical guidance. A logic model was then used to design a care pathway (Appendix S1) [26]. A first draft of the checklist incorporated findings from the focus groups and a literature and guidelines search, and the original format was based on that of a previous MBRRACE confidential morbidity review investigating the effect of ethnicity on maternity care [27]. In accordance with NHS guidelines, ‘interpreter involvement’ was defined as the use of professional interpreting services either in‐person or via video or phone call. Informal assistance from family members was not recognised as official interpreting support [28]. The first draft of the checklist was reviewed by a multi‐professional steering group of 42 members. Two separate focus groups for migrant women who spoke English as an additional language and those from minority ethnic backgrounds were used to refine the language support and racism aspects of the checklist. This was subsequently reviewed by the NHS Race and Health Observatory and cross‐checked against other research and report findings [29, 30, 31, 32, 33]. A focus group of professional stakeholders and service users met to review a list of prompts suggesting complex social factors may be present. The complex social factors chosen were based on UK National Institute for Health and Care Excellence guidance for the care of women experiencing complex social factors and a list of factors identified in a joint report by two charities supporting women with multiple disadvantage in pregnancy in the UK [34, 35]. The final draft of the main checklist and the accompanying prompt lists were sent to all stakeholders, including service users, for final comments.

### 2.5 Systematic Case Note Review

After finalising the check list, the systematic medical record review was conducted to describe the care received and generate hypotheses on how to improve maternity care for recently arrived migrant women in the UK. The main (KS) and second reviewer (NV) piloted the checklist and accompanying prompt lists with two sets of records. Any improvements or corrections were made, and both reviewers agreed on the final approach. The main reviewer completed the checklist on all medical records, whereas the second reviewer assessed a 20% sample. They met for a consensus meeting, and it was agreed in advance that if their responses differed substantially (more than 15%) the second reviewer would extract data from another 20% sample. This approach would be repeated until both reviewers were satisfied that the reviews were systematic and reliable. Following extraction of data from the first 20% of records, the data were consistent, and the main reviewer extracted the rest of the checklist information. Stakeholders and service users later met to discuss key findings. Definitions and clinical cut‐offs are detailed in Appendix S2.

### 2.6 Potential Areas for Improving Maternity Care for Recently Arrived Migrant Women in the UK

To generate hypotheses about areas of care that could be improved, the care received was categorised in the following manner. If less than 50% of women received the checklist‐recommended care this was considered as an ‘Immediate Improvement Area’. If 50%–75% of women received the checklist‐recommended care this was considered as an ‘Urgent Improvement Area’. If greater than 75% of women received the checklist‐recommended care this was considered a ‘Desirable Improvement Area’.

## 3 Results

The co‐produced recently arrived migrant maternity care pathway resulting from the logic model exercise with service users is presented in Figure 1 and Appendix S2. Five key care domains were identified: overall quality of care; interpreting services, antenatal care, intrapartum care, and postpartum care. The prompts of factors suggesting discrimination due to ethnicity or migration, and factors relating to complex social factors are presented in Appendices S3 and S4.

**FIGURE 1:**
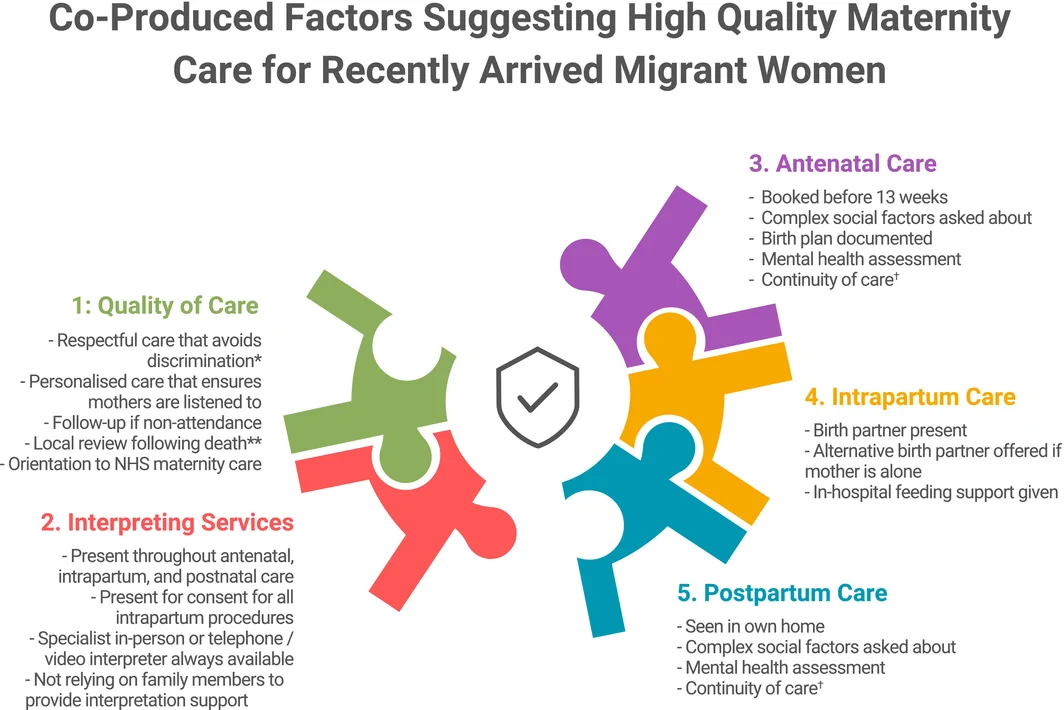
Co‐produced maternity care pathway for recently arrived migrant women in the UK. *Respectful care that avoids discrimination was defined as care that ensures women are treated equally regardless of protected characteristics [36], including their ethnicity and immigration status. **Not all deaths are eligible for local review e.g., deaths of women that never received NHS care. ^†^Continuity of care may be provided by midwifery and/or obstetric staff. It may not be solely delivered by one person, but could be delivered by a small group of known clinicians in a team.

### 3.1 Maternal Demographic Characteristics, and Past Medical and Obstetric History

Between 2012 and 2021, 39 recently arrived migrant women died in the UK during pregnancy or up to six weeks after birth (Table 1). The average age at death was 30 years and 21% (*n* = 8) were aged 35 or older. 41% (*n* = 16) of women were born in a high‐income country, 54% (*n* = 21) in a middle‐income country, and 5% (*n* = 2) in a low‐income country. Almost one third (28%, *n* = 11) of women were pregnant on arrival in the UK. Most women (49%, *n* = 19) were of ‘White Other’ ethnicity, followed by Black African ethnicity (23%, *n* = 9). Approximately half of women had pre‐existing medical problems (54%, *n* = 21), and three (8%) had a pre‐existing mental health diagnosis. Most women died in the antenatal period (59%, *n* = 23), and the most common reported cause of death was cardiac disease (23%, *n* = 9) followed by coincidental death (a death from an unrelated cause which happens to occur in pregnancy or puerperium e.g., road traffic accident) (21%, *n* = 8) (Table 1).

**TABLE 1:** Maternal demographic characteristics, and past medical and obstetric history. (*N* = 39 women).

	*n* (%)*
MaternMaternal age at death (years)	
Less than 24	7 (18%)
25–34	24 (62%)
35–39	5 (13%)
40 or more	3 (8%)
World Bank income category of country of origin	
Low	2 (5%)
Lower‐Middle	17 (44%)
Upper‐Middle	4 (10%)
High	16 (41%)
Number of months in UK prior to death	
0–1 months	5 (13%)
2–6 months	6 (15%)
7–12 months	17 (44%)
13–24 months	11 (28%)
Visitor status	
Short‐term/Tourist visa (< 6 months)	3 (8%)
Long‐term visa	25 (64%)
Not recorded	11 (28%)
Immigration status**	
EU Citizen/Resettlement Visa	13 (33%)
Student	4 (10%)
Refugee/Asylum Seeker	4 (10%)
Spouse/Partner of UK Citizen	3 (8%)
Known to be undocumented	2 (5%)
Tourist/Short term visitor	2 (5%)
Not recorded	11 (28%)
Ethnicity	
White Other	19 (49%)
Black African	9 (23%)
Asian Pakistani	5 (13%)
Arab	3 (8%)
Asian Other	3 (8%)
Living with/in contact with partner	
Yes	34 (87%)
No	5 (13%)
Pregnant on arrival to the UK	
Yes	11 (28%)
No	27 (69%)
Not recorded	1 (3%)
Trimester of death	
1st trimester	2 (5%)
2nd trimester	8 (21%)
3rd trimester	13 (33%)
Postpartum	16 (41%)
Cause of death^a^	
Cardiac disease	9 (23%)
Coincidental	8 (21%)
Infection indirect	7 (18%)
Death from early pregnancy causes	3 (8%)
Neurology	3 (8%)
Other direct	3 (8%)
Psychiatric causes	2 (5%)
Infection direct	2 (5%)
Other indirect	2 (5%)
Not recorded	3 (8%)
Pre‐existing medical problems (including obesity)	
Yes	21 (54%)
No	15 (39%)
Pre‐existing mental health diagnosis	
Yes	3 (8%)
No	27 (69%)
Not recorded	9 (23%)
Number of previous pregnancies beyond 22 weeks	
0	13 (33%)
1 to 2	9 (23%)
≥ 3	6 (15%)
Not known	11 (28%)

* Percentages may not add to 100 given rounding.

** Immigration status definitions: EU Citizen/Resettlement Visa: European Union citizen or given a longer term visa as part of the EU Settlement Scheme. Student: possessing a student visa. Refugee/Asylum Seeker: has applied for asylum in the UK (asylum seeker) or been granted asylum (refugee). Spouse/Partner of UK Citizen: allowed to live in the UK as their spouse/partner is a UK citizen; Known to be undocumented: a person who has not been granted authorisation to enter, live or work in the UK; Tourist/Short term visitor: In the UK for holiday/family visiting purposes.

^a^ MBRRACE‐UK cause of death definitions (World Health Organisation 2010): Direct: Maternal deaths resulting from obstetric complications of the pregnant state (pregnancy, labour and puerperium), from interventions, omissions, incorrect treatment or from a chain of events resulting from any of the above. Indirect: Maternal deaths resulting from previous existing disease, or disease that developed during pregnancy and which was not the result of direct obstetric causes, but which was aggravated by the physiological effects of pregnancy. Coincidental: Maternal deaths from unrelated causes which happen to occur in pregnancy or the puerperium.

### 3.2 Summary of Care Received in Most Recent Pregnancy

Almost one quarter of women missed planned appointments (23%, *n* = 9), and only one third of those were followed up. Improvements to the pathway of care were needed for over one third of women, e.g., being unable to move to labour ward due to lack of free beds or not being referred to a specialist clinician (39%, *n* = 15). Two fifths of women's records (41%, *n* = 16) suggested evidence of discrimination or racism as assessed with the use of the discrimination or racism prompts (Appendix S3). NHS maternity charging status was not recorded for most women (62%, *n* = 24). Three (8%) women were recorded as being liable for NHS charging in the medical records; one was recorded as actually having been charged, but it was not clear if the other two were charged. None of these women returned for follow‐up care after they were told they were eligible for charging. Results are presented in Appendix S4.

### 3.3 Language Interpretation in Most Recent Pregnancy

Amongst women needing an interpreter for antenatal care, 42% *(n* = 8/19) had a professional interpreter present and of these interactions, two thirds were via a live professional on video or telephone and the remainder were in‐person. Of those that received intrapartum care and required an interpreter, 19% (*n* = 3/16) had an interpreter and all had an in‐person professional interpreter present. Amongst women who received postpartum care and needed an interpreter, none (0%, *n* = 0/9) received formal interpreting support. The results are presented in Appendix S4.

### 3.4 Antenatal Care in Most Recent Pregnancy

Thirty women received NHS antenatal care (ANC) in their most recent pregnancy. Of those who did not arrive in the UK pregnant, 19 (70%, *n* = 19/27) had their first ANC appointment after 13 weeks. Four women (13%) experienced continuity of midwifery or obstetric care. 18 women (60%) had their mental health assessed at any point in ANC, and most (78%, *n* = 14) were assessed using a validated mental health screening questionnaire. Only one woman was newly identified as potentially having a mental illness. Three women (10%) disclosed they were a current victim of interpersonal/domestic violence. Resulting action (e.g., referral to local support agencies) was not evidenced in any of their medical records. When considering the routine assessment of complex social factors in ANC, only 10% (*n* = 3) of ANC records reviewed prompted maternity care providers to ask about financial need, 17% (*n* = 5) whether a woman is living in asylum/refugee accommodation, 28% (*n* = 11) about interpersonal/domestic violence, and none routinely prompted to ask women if they were a victim of trafficking or modern slavery. Results are presented in Appendix S4.

### 3.5 Intrapartum Care in Most Recent Pregnancy

Thirty women received intrapartum care. The most common mode of birth was Caesarean section (57%, *n* = 17). Five women (17%) had an induction of labour, 18 women (60%) were admitted to critical care, and two (7%) had a stillborn infant. Amongst those for whom it was applicable to have a birth partner present (e.g., not requiring an emergency Caesarean section with general anaesthetic), 14 (58%) had a birth partner, three (8%) did not, and the presence of a birth partner was not recorded for seven (29%) women. Amongst those who did not have a birth partner, an alternative birthing partner was not recorded as having been offered. Results are presented in Appendix S4.

### 3.6 Postpartum Care in Most Recent Pregnancy

Eight women were eligible for postpartum maternity care. Four (50%) had their mental health assessed postnatally, two were assessed using a formal validated mental health screening questionnaire, and one woman was identified as being at risk of having a mental illness; it was not clear whether or not she was referred for support. Two women (25%) had continuity of midwifery/obstetric care in the postpartum period. It was not possible to make a reliable assessment of the number of postpartum visits received as women were often readmitted to hospital in the early postpartum period. Results are presented in Appendix S4.

### 3.7 Suggested Areas for Improvement in the Maternity Care of Recently Arrived Migrant Women Who Died

Potential areas for improvement in the care of recently arrived migrant women were hypothesised by assessing the care received and comparing it to the care outlined in the five domains of the migrant maternity care checklist (Figure 2). The hypothesised immediate improvement areas were: respectful care that avoids discrimination; follow‐up after non‐attendance at appointments; access to formal interpreting services; support to access antenatal appointments before 13 weeks; assessing the presence of complex social factors; discussing and recording a birth plan; offering a birth partner if the mother is alone; in‐hospital feeding support; and continuity of postpartum midwifery care.

**FIGURE 2:**
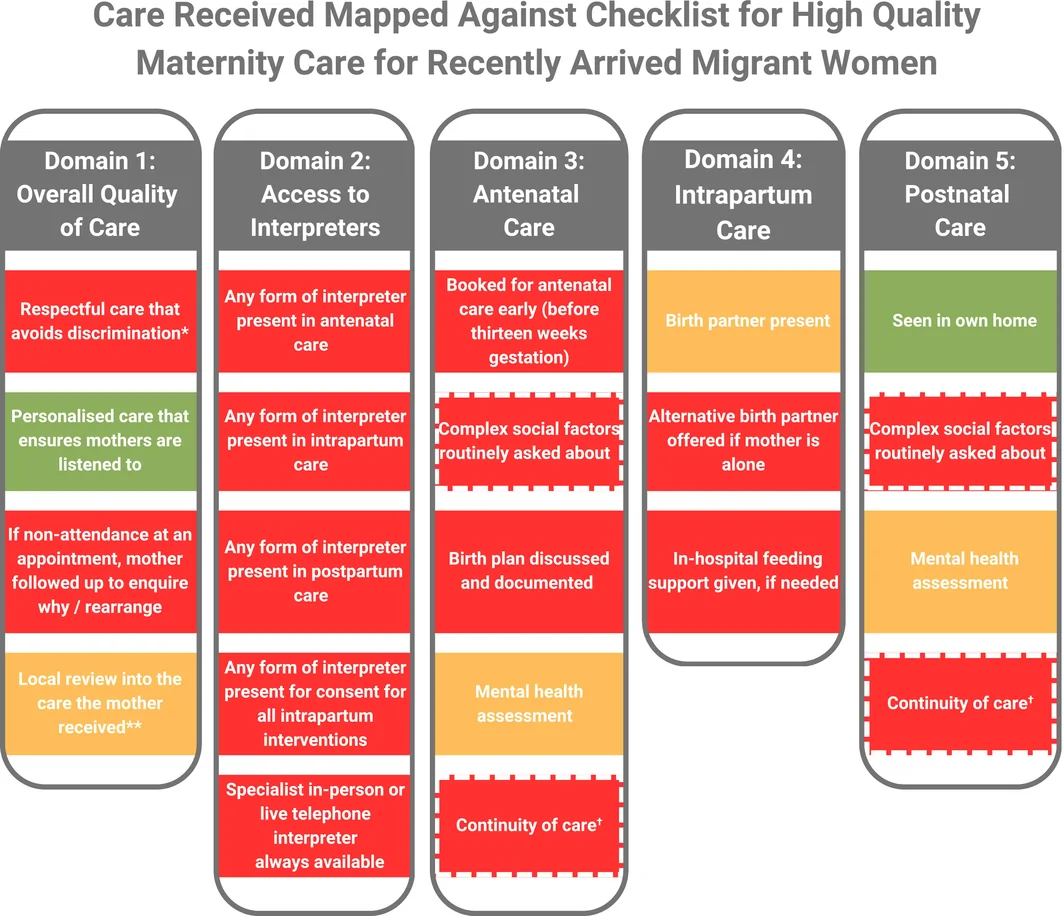
Care received mapped against the checklist for high quality maternity care for recently arrived migrant women. If less than 50% of women received the checklist‐recommended approach this was categorised as an ‘Immediate Improvement Area’ and coloured red. If 50%–75% of women received the checklist‐recommended standard approach this was categorised as an ‘Urgent Improvement Area’ and coloured amber. If greater than 75% of women received the checklist‐recommended standard approach this was categorised as ‘Desirable Improvement Area’ and coloured green. The dashed boxes indicate that these aspects of care were difficult to assess from the notes. For both complex social factor assessment and continuity of care may have occurred but not have been recorded in the notes. *Respectful care that avoids discrimination was defined as care that ensures women are treated equally regardless of protected characteristics [36], including their ethnicity and immigration status. **Not all deaths are eligible for local review e.g., deaths of women that never received NHS care. ^†^Continuity of care may be provided by midwifery and/or obstetric staff. It may not be solely delivered by one person, but could be delivered by a small group of known clinicians in a team.

## 4 Discussion

### 4.1 Main Findings

This national confidential enquiry identified that 39 recently arrived migrant women died during or up to six weeks after pregnancy in the UK between 2012 and 2021. Using a co‐produced recently arrived migrant maternity care checklist to describe and categorise care received by this group of women, it was hypothesised that several areas of improvement exist: timely antenatal care before 13 weeks, follow‐up after missed appointments, access to interpreters, assessment of complex social factors, and continuity of midwifery care. Caution is required around the exact point estimates calculated given the small number of women included and the hypothesis‐generating nature of this paper. In addition, we were reliant on what was recorded in the medical records and absence in medical notes cannot be equated to absence in practice. One of the experts by experience who co‐designed this research has reflected on its findings; her insights add lived experience context to the report findings (Box 1).

BOX 1 **Observation on these Findings from a Lived Experience (Service User) Perspective.**

**Table:** 

*I am a migrant mother who had a baby in the UK NHS maternity system. I often felt things weren't explained to me, and my consent wasn't fully sought for various interventions including induction. The findings from this review are very sad but not shocking to me, and they represent an urgent call to action*. *Migrant mothers often struggle to navigate maternity health in the UK due to stark differences between the healthcare systems of their home countries and the NHS. Limited understanding of how UK maternity care operates, combined with cultural differences in pregnancy, childbirth, and help‐seeking, can create confusion and anxiety. Many mothers also lack a support system, having left behind extended family networks central to their wellbeing. Mental health conditions during pregnancy are often viewed through cultural lenses, where distress is rooted in isolation and unmet basic needs rather than recognised as clinical issues. This mismatch can hinder engagement with services*. *Supporting migrant mothers requires a two‐way approach. Migrants need help understanding how the NHS works, what maternity services provide, and what to realistically expect. At the same time, NHS maternity teams, midwives, and health visitors need deeper awareness of migrant experiences such as migration stress, emotional and physical isolation, financial strain, childcare worries, and fears, such as misconceptions about children's services. Meeting foundational needs first can create safety, trust, and improved outcomes for migrant mothers and their babies*.

### 4.2 Strengths and Limitations

Before this study, there had been no detailed assessment of recently arrived migrant women who died in the UK during the peripartum period. Unlike the recent MBRRACE morbidity review, which focused on women with language barriers who survived, this study includes all such deaths over a decade. Case finding was comprehensive, and methods involved two independent reviewers and a stakeholder panel, including service users, to design the recommended migration maternity care checklist and interpret findings.

Key limitations include the small sample size of 39 women with varying migration backgrounds, causes of death, and care pathways. In addition, the sample is highly heterogenous and incorporates 39 women with varying migration backgrounds, causes of death, and care pathways which restricts the study to descriptions of characteristics and care. This limits the ability to draw conclusions specific to this population. For example, whilst the need for interpreter services is likely specific to recently arrived migrant women, other findings, such as the need to identify complex social circumstances and evidence of bias in care, have been identified in other confidential enquiries of women that die during pregnancy in the UK and may reflect wider system challenges [23, 37, 38, 39]. The study is also limited by a lack of comparator population. This is due to the lack of national NHS data on migration which would enable us to identify a suitable sampling frame to identify comparator women. However, we have tried to compare our findings with previous MBRRACE maternal mortality enquiries and the UK's general maternity population.

The co‐creation of the checklist with clinicians, academics, and migrant women is a strength of the study. It represents an aspirational list of practices that those who helped develop it deem to represent high quality maternity care for recently arrived migrant women. However, it has not been tested in a real‐world clinical context or externally validated. Many parts of the checklist are not easy to implement in the real‐world clinical context, particularly given the staff shortages, limited access to interpreting services, and high complexity and patient turnover in the current NHS maternity system. Further qualitative and quantitative research is required to better understand the life and care experiences that are specific to recently arrived migrant women, and the interventions or models of care that may be most effective at improving maternity outcomes.

This study is reliant on documentation within medical records, which made assessing some aspects of care such as discrimination challenging. We cannot conclude that absence of documentation in the medical records is truly absence in practice, for example continuity of care, birth partner support or follow up after non‐attendance may not always be documented. The review also spans 10 years, including the COVID‐19 pandemic, so trends over time must be considered.

### 4.3 Interpretation

The 2024 MBRRACE‐UK review assessed maternity care received by recently arrived migrant women whose preferred language was not English and who experienced morbidity but who did not die [21]. Similarly to these findings, there was over‐reliance on family members or Google Translate for interpreting, poor documentation of language needs, and lack of formal interpreters for consent. In this study, most women who needed an interpreter did not receive one in antenatal, intrapartum, or postnatal care. This was most obvious in postnatal care where none of the women who needed it received professional interpreting support. During the review, most interpreting support was provided by friends and family. However, national guidance states that independent professional interpreters should be used throughout maternity care, and some research suggests that women are at increased risk of harm when family members interpret during maternity care [21, 22]. In this review, two fifths of women potentially experienced some form of discrimination or racism due to their ethnicity or their migration status. Examples of potential discrimination or racism included incorrect and inconsistent ethnicity records, not offering support with immigration concerns, and not offering a female physician despite maternal request. The recent MBRRACE review also found that women may have experienced microaggressions and discrimination in their care [21]. These findings suggest the need for action to address racism and discrimination in maternity care, particularly in relation to correct ethnicity recording which is vital to ensure improved outcomes for patients from minority ethnic backgrounds [40, 41, 42] Additionally, approximately 10% of the women in this study self‐discharged from care. This is higher than the national average of 3%, and although we do not know why they self‐discharged, it may be due to unmet needs, lack of trust in services, fears of NHS charging, or dissatisfaction with care [43].

In this study, we found that complex social factors were often not recorded as being assessed, and that most medical records did not offer routine prompts for maternity staff to ask about their presence. Whilst this may reflect practice that occurred but was not documented, national guidance stresses the need for clinical staff to assess the presence of complex social factors and to offer referrals for support [44, 45]. Similarly to these findings, the MBRRACE morbidity review highlighted significant gaps in the assessment of and support for women with complex social factors [21, 44]. The MBRRACE migration morbidity review also highlighted that women who did not attend appointments were not always followed up and supported to attend in future [21]. Similarly, we found that one third of women who did not attend appointments had a subsequent documented contact to enquire why or rearrange the appointment, although further contacts could have been undocumented.

In both this review and the recent MBRRACE review, one third of women arrived in the UK whilst pregnant which suggests the need for interventions to improve timely access to NHS antenatal care for newly arrived migrant women [21]. Only three women were identified as being liable for NHS maternity care charging in this review, but research suggests this policy may impact women's trust in maternity services and influence care‐seeking behaviour [17, 19, 20]. Of the women whose care we reviewed, most were not assessed by their health professional for eligibility to free NHS maternity care. This is not considered within their professional scope of practice and may reflect midwives' concerns of the impact on women's trust in services and quality of care [17, 19, 20]. However, in this review some hospitals had an automatic referral system for midwives to flag when someone is likely to be eligible for NHS charging. The recent MBRRACE review found that asylum seekers were being moved close to their due date or early in the postpartum period which goes against national guidance [21, 46, 47]. The asylum seekers in this review were not recorded to have received any additional support from their midwifery team, and one was moved late in pregnancy.

The average age of women who died is not stated in MBRRACE reports, but recent trends show the majority of women who die are over 30 years old [23]. The average age of mothers in this study was potentially younger than that (30 years), but this should be interpreted with caution given low numbers. In this review, most deaths occurred antenatally, but nationally most maternal deaths occur in the postpartum period [33, 34, 35, 36, 37, 38, 39, 40, 41, 42, 43, 44, 45, 46, 47, 48]. The reasons behind this difference are unclear. In the 2020–22 MBRRACE population‐level mortality report, 74% of women had a caesarean section (CS); in this review, 57% of women underwent a Caesarean birth [23]. International systematic review findings suggest certain migrant women are at higher risk of CS, perhaps due to poorer access to antenatal care, language barriers, poorer maternal health, or maternal request [49]. In the most recent MBRRACE mortality report, approximately 80% of women from the general population had their first antenatal appointment before 13 weeks of pregnancy [23]. In this study only 30% of women had their first antenatal appointment before 13 weeks [50]. This suggests the ongoing need for action, including outside of maternity services, to ensure early engagement for newly arrived women in the UK.

## 5 Conclusion

This study has described care and generated hypotheses about potential improvements to care for recently arrived migrant women in the UK maternity system. Potential areas for improvement include access to professional interpreting services; access to first antenatal appointments before 13 weeks; follow‐up after non‐attendance at appointments; assessing for the presence of complex social factors; continuity of postpartum midwifery care; and improvements in the delivery of respectful care that avoids discrimination. The sample in this study is small and highly heterogeneous; therefore, we cannot conclude which improvements are truly specific to improving care for recently arrived migrant women. However, many of the suggested improvements align with findings from other confidential inquiries in maternity care, and therefore could potentially improve care for other groups of underserved women in the NHS maternity system. There is an urgent need for population‐level data on maternity outcomes amongst all migrant women giving birth in the UK to fully understand where care should be improved. As the UK's maternity population becomes increasingly diverse, the maternity system must adapt to provide the best possible care to newly arrived migrant women.

## Author Contributions

Kerrie Stevenson, Nicola Vousden, Kemi Ogunlana, Nirupama Prabhakar, Sara Kenyon, Brenda Kelly, Allison Felker, Robert W. Aldridge, Marian Knight, and Fiona Stevenson contributed to the conception of the review. Kerrie Stevenson, Sara Kenyon, Brenda Kelly, Nicola Vousden, Kemi Ogunlana, and Marian Knight contributed to the methods. Kerrie Stevenson and Nicola Vousden completed the case note review, and Kerrie Stevenson completed the basic characteristics analysis. Marian Knight provided supervision. Kerrie Stevenson drafted the paper, with contributions from Marian Knight, Nirupama Prabhakar, and Nicola Vousden. All authors contributed to the data interpretation and review and editing. All authors had full access to all the aggregated data in the study (case notes viewable only to the two main reviewers) and had final responsibility for the decision to submit for publication.

## Funding

KS is supported by an NIHR Doctoral Fellowship (NIHR302577), this study is part of a peer‐reviewed fellowship. The views expressed are those of the authors and not necessarily those of the NIHR or the Department of Health and Social Care (DHSC). This work is supported by the NIHR Great Ormond Street Hospital Biomedical Research Centre (GOSH BRC). The views expressed are those of the author(s) and not necessarily those of the NHS, the NIHR or the DHSC. SK is part funded by the Applied Research Collaboration West Midlands. MK and SK are NIHR Senior Investigators. The views expressed are those of the authors and not necessarily those of the NHS, the NIHR, or the Department of Health and Social Care. The work is also supported by the Healthcare Quality Improvement Partnership (HQIP). The funders had no role in study design, data collection, data analysis, data interpretation, or writing of the report.

## Ethics Statement

Identifiable information about maternal deaths occurring in England and Wales is collected without consent with approval of the Secretary of State for Health and Social Care under Section 251 of the NHS Act 2006 (15/CAG/0119). Data from Scotland is collected by MBRRACE‐UK without consent with approval from the Public Benefit and Privacy Panel for Health and Social Care (PBPP 2223–0101). Identifiable information is not provided by Northern Ireland. The legal basis for this activity is Article 6 (1)(e) and Article 9 (2)(i) under the General Data Protection Regulation. Approval for use of the data was obtained via the Healthcare Quality Improvement Partnership Extended Output Process.

## Conflicts of Interest

The authors declare no conflicts of interest.

## Supporting information


**Appendix S1:** Co‐Produced Prompt of Factors which may be Indicative of Discrimination during Maternity Care.
**Appendix S2:** Co‐Produced Prompt of Factors Suggesting Complex Social Factors are Present.
**Appendix S3:** Detail on the definitions and clinical cut‐offs used in the ‘Gold Standard Care’ checklist.
**Appendix S4:** Additional results from the case note review.

## Data Availability

The data that support the findings of this study are available on request from the corresponding author. The data are not publicly available due to privacy or ethical restrictions.
