# Supplementary material for: Care Received by Migrant Women Who Died During or After Pregnancy: A National Confidential Case Note Review
Source: BJOG. 2026 Jun 23;133(12):2159–69. doi: 10.1111/1471-0528.70288 (PMC13659352; doi:10.1111/1471-0528.70288)
Supplement: Supplementary file 1 [file BJO-133-2159-s001.docx]

**APPENDICES**

**Appendix 1: Co-Produced Prompt of Factors which may be Indicative of Discrimination during Maternity Care**

**Factor (with examples)**

**RELATING TO BEING FROM AN ETHNIC MINORITY GROUP**

***Demeanour / Attitudes / Respect***

- Documented descriptions of mother or her family being angry or complaining about the care received.
- Mother described as agitated and being told to calm down.
- Medical staff documenting they have reassured the mother but taken no action to address her concerns which may suggest her concerns are belittled.
- Agitation attributed to mental health problems.
- Failure to respect maternal wishes surrounding childbirth, e.g. refusal to support homebirth or delivery in a midwifery-led unit without clear clinical reasons, or not providing further discussion opportunities if a choice outside of guidance is preferred.
- Documented discussion of ability to cope with pain / perceptions of pain in childbirth or pregnancy.
- Discussion of mother’s ethnicity and her childbirth, e.g. how perceived physical differences due to ethnicity may impact her birth.
- Not asking about or refusal to allow mothers to engage in cultural or religious practices during maternity care (note some practices may be harmful, and an informed and sensitive conversation about these should not be considered indicative of discrimination due to ethnicity).
- Derogatory or belittling discussion of cultural practices in pregnancy, intrapartum, or postpartum such as maternal confinement, family involvement, religious practice, circumcision, herbal remedies, wish to keep the placenta, or traditional medicine.
- Refusal to allow women to eat food brought in by family and friends.
- Refusal to respect wishes to avoid nudity or removal of headscarves in front of male staff or staff in general.
- Threats of / actual referral to local authority children’s social care teams / safeguarding on the grounds of practice or behaviour that may be linked to ethnicity, or cultural, or religious practice.

***Quality of Care Offered / Provided***

- No documented discussion of birth preferences or a birth plan.
- If gaps in care identified, e.g. missed appointments, appropriate steps should have been taken to understand why gaps occurred. For example, if there was non-attendance at clinic, was there contact with pregnant woman to understand why and to help them engage in care?
- Documented request for treatment or pain relief which was not acted upon or delayed.
- Mother being given medication or intervention with no discussion of other options or consent in notes.
- Induction of labour without consent being documented.
- Documented discussion of ethnicity in relation to decision to offer pain relief or intervention or not e.g. her ability to cope with pain due to her ethnic background.
- Women receiving inadequate pain relief for sickle cell crises.
- Insertion of postpartum intrauterine contraception device without consent being recorded.
- Non-invasive drug testing in urine of neonate when no obvious indication, or drug testing the mother with no obvious indication or consent.
- Failure to refer or ignorance of relevant agencies/charities for appropriate support, if needed.
- Failure to provide female healthcare staff when mother has requested this.
- Failure to provide care / reviews for long periods, e.g. left overnight in pain with no support.
- Extra ‘ethnicity scans’ being offered, e.g. reason cited being that women from an ethnic minority group are at higher risk of death or complication in pregnancy.

***Consent / Communication***

- Sense from the notes that maternity staff are making clinical decisions for mothers without discussion or taking consent.
- Ignoring concerns or complaints from mothers or family members.
- Lack of documented family updates / discussions which suggests lack of communication between patient and staff.
- Lack of documented consent for examinations, interventions in labour care e.g. pelvic examination, extra monitoring in labour, oxytocin use.
- Not explaining what is happening in a way the mother understands, particularly when things deviate from the norm, e.g. using medical terms or abbreviations and not explaining the meaning.
- Failure to provide educational materials / literature on warning signs.

***Language Interpretation***

- Not arranging formal interpreting support in for a pregnant person who is not proficient in English.
- Lack of respect for maternal request for an interpreter in all appointments, especially refusal to arrange a female interpreter (unless practically impossible to do so).
- Offering an interpreter in a similar dialect/language to mother’s primary language, but not offering the patient’s actual language.
- Refusal to arrange or communicate via an interpreter, especially when consenting for medical procedures.
- Not allowing sufficient time for appointments with interpreter.
- Choosing to communicate via family members when mother requires a formal independent interpreter.
- Not supplying patient information in primary language of the pregnant person where patient information is known to be available in their language.

***Documentation***

- Use of racist terms in medical records, e.g. ‘ethnic mother’, ‘foreign lady’, ‘coloured’, 'Afro-Caribbean’.
- Incorrect or poorly informed recording of ethnicity and nationality in the notes. Women’s ethnic group and origins described in varying ways throughout their notes.
- Documented concerns or a formal complaint from a mother or family from a minority ethnic background which suggests she may have received differential care.
- Only offering medical records through digital format, to which not all women will have access to or understand.
- Large or obvious omissions in maternity records.

***References / Mistreatment Relating to Skin Colour***

- Documented discussion of baby’s skin colour, name, health, size, or behaviour (likely due to ethnic background).
- Failure to recognise conditions in mother and baby due to skin colour e.g. sepsis or jaundice.
- Asked about NHS charging or immigration status based on skin colour.

**RELATING TO MIGRATION**

***Discrimination due to Immigration Status***

- Staff employing invasive questioning / approaches / techniques to determine mother’s immigration and NHS charging status.
- Staff using phrases in notes such as ‘criminal migrant’ or ‘illegal migrant’ in notes.
- Documented unprompted discussion asking when mother plans to return home (assumption from staff that the UK is not home).
- Discharging women to a different address at the request of the Home Office without checking out the accommodation is suitable for the woman and newborn.

***Lack of Support in Orientating to NHS System***

- Not explaining the NHS maternity system to newly arrived migrants.
- Placing the onus on women to arrange antenatal appointments themselves and giving no other option to newly arrived migrant women.

***Proactive Pursuit of NHS Charging***

- Documented discussion with mother explaining she is not eligible for pregnancy care unless she pays (official NHS guidance is clear that maternity care should never by refused or withheld, regardless of ability to pay).
- Staff reporting mother to hospital Overseas Visiting Team in relation to chargeability for care (as this extends out with professional scope of care).
- Trust’s Overseas Visiting Team visiting mother in hospital and asking about eligibility for NHS care, or asking for a down payment on care before further treatment offered (official NHS guidance is clear that maternity care should never by refused or withheld, regardless of ability to pay).
- Trust’s Overseas Visiting Team arriving on ward demanding payment, e.g. arriving with card machine.
- Mother restricted from leaving the ward or from being discharged until she has seen the hospital Overseas Visiting Team.
- Hospital Overseas Visiting Team attend the ward at inappropriate times e.g. quiet times or when mother is feeding her newborn.
- Mother being sent an invoice for NHS care in pregnancy e.g. at booking and expressing concern to maternity staff.
- Comments in relation to the cost of care, ability to pay, impact on services.

***Proactive Pursuit of Immigration Enforcement***

- Staff reporting mother to immigration authorities when suspecting she has an undocumented immigration status.
- Documented discussion of immigration status, with staff warning mother they are at risk of being deported and / or without offering any legal advice or support.

**Appendix 2: Co-Produced Prompt of Factors Suggesting Complex Social Factors are Present**

**Factor (with examples)**

**Significant financial need**

- Mother asking about benefits / financial / debt support
- Mother asking about access to free baby supplies / foodbanks
- Concerns flagged by staff e.g. inadequate new baby equipment post-delivery

**Insecure housing**

- Experiencing or at risk of homelessness e.g. temporary accommodation such as a B&B, rent arrears, ‘sofa surfing’, rough sleeping
- Living in an overcrowded home / unsafe home e.g. damp
- Mother asking about support with housing
- Concerns flagged by staff on home visits

**Living in asylum hotel / accommodation**

- Extractor should check mother’s home address to see if it is known to be an asylum hotel / accommodation
- Discussion of poor access to cooking facilities / private spaces at home as in shared asylum accommodation

**Moved by Home Office during pregnancy**

- Note a change in address from one hotel / accommodation to another and known asylum seeker
- Mother asking midwife for support with writing a letter to the Home Office to tell them she is pregnant and asking not to be moved

**At risk of deportation**

- Known to be an undocumented migrant or known to have had asylum application rejected
- Mother tells staff she is concerned about being deported or has been told she will be by the authorities
- Mother is trying to evade deportation

**In immigration detention facilities**

- This is the deprivation of liberty or confinement of an asylum seeker in a secure place
- Mother living in immigration detention; including prisons, holding centres, or purpose-built detention centres

**Criminal justice involvement**

- Residing in prison or probation service involvement
- Mother discloses police presence or investigations

**Social services involvement**

- Family support worker involved
- Child in care of social services antenatally
- History of social service involvement with family / previous pregnancies

**Interpersonal / domestic violence**

- Staff expressing concerns about interpersonal / domestic violence, or referring to police, social services, and domestic violence charities
- Mother discloses abuse towards her or other children
- Multiple physical injuries which staff report have been covered up or explained away
- Attending appointments with a controlling partner who refuses to leave during consultations

**Victim of trafficking / modern slavery**

- Mother discloses experiences to staff
- Mother giving vague or contradictory information about where she lives or works, or why she came to the UK
- Staff expressing concerns due to signs of withdrawal, physical neglect, and concern about trafficking
- Attending appointments with a controlling person who seems to be unrelated, or attending with multiple different people over time
- Mother has untreated long-term physical injuries

**Appendix 3: Detail on the definitions and clinical cut-offs used in the ‘Gold Standard Care’ checklist**

Data for each item on the checklist for each case was inputted into Microsoft Excel, and frequencies and percentages calculated^1^. Some free text data were collected to aid understanding of a woman’s care, or for the benefit of reviewers, particularly when assessing exposure to discrimination in maternity care. Ethnicity categories were chosen to reflect those in the 2021 UK census.^2^ World Bank country income categories were used to classify maternal country of birth into low-, middle-, or high-income countries.^3^ ‘Potential delays to care’ were defined as those identified by local or national review bodies, or the likely presence of a delay in care as assessed by the reviewers. ‘Mother’s concerns raised and not met at any point in care’ was defined as those identified by local or national review bodies, or the presence of an instance of the mother’s concern being documented without corresponding documented action as assessed by the reviewers. An assessment of whether complex social factors were routinely prompted and asked about in antenatal care was conducted by reviewing maternal case notes such as the presence of tickboxes or prompts to maternity care providers. Complex social factors were defined using the National Institute for Health and Care Excellence (NICE) guidelines for the care of pregnant women.^4^ Late antenatal care booking was defined as not having been seen for a booking appointment by thirteen weeks gestation as defined by the NHS key performance indicator for antenatal care booking, for those that were in the country prior to this gestation.^5^ Continuity of midwifery or obstetric care was defined as a known midwife / obstetrician or small team of midwives / obstetricians whom the woman sees regularly throughout the antenatal care period.^6^ Birth plan documentation was only expected from thirty-four weeks gestation as per NICE antenatal care guidance.^7^ Mental healthcare assessment was conducted using NICE guidelines, which recommend routine mental health assessment in both the antenatal and postnatal periods and suggest using the Whooley questions, or equivalent.^8^

**Appendix 4: Additional results from the case note review**

| **Appendix 4.1: Summary of care received in most recent pregnancy. (N=39 women).** | |
| --- | --- |
|  | **n (%)*** |
| Improvements needed to pathways of care (i.e. instance of a women’s care being postponed, slowed down, or cancelled by an NHS provider. Excludes instances where women did not attend or cancelled appointments)  Yes  No  Nature of improvement needed (n=15)  Earlier referral to specialist services  Earlier receipt of treatment in busy unit  Patient discharged whilst still unwell | 15 (39%)  24 (62%)  7 (47%)  5 (33%)  3 (20%) |
| Recorded in the medical records as being liable for NHS charging in this pregnancy  Yes  No  Not recorded  Not applicable | 3 (8%)  10 (26%)  24 (62%)  2 (5%) |
| Patient missed at least one planned appointment (i.e. Did Not Attend – ‘DNA’)  Yes  No  Not applicable  If non-attendance, were they followed up? (n=9)  Yes  No  Not recorded | 9 (23%)  24 (62%)  6 (15%)  3 (33%)  4 (44%)  2 (22%) |
| Self-discharge at any point in care  Yes  No | 4 (10%)  35 (90%) |
| Mother’s concerns raised and not documented to be met at any point in care  Yes  No  Not applicable | 5 (13%)  32 (82%)  2 (5%) |
| Potential evidence of discrimination  Yes  No  If yes, what form of discrimination was suspected?** (n=16)  Incorrect / inconsistent ethnicity records  Not offered social support despite requesting | 25 (64%)  14 (36%)  14 (70%)  4 (20%) |
| * Percentages may not add to 100 given rounding.  ** Some women are counted in more than one category, and examples with small numbers of women are narratively presented in the discussion. | |

| **Appendix 4.2: Language interpretation in most recent pregnancy.** | |
| --- | --- |
|  | **n (%)*** |
| *Needing an interpreter for Antenatal Care (ANC) (n=19):*  Interpreter present in ANC?**  Yes  No  For those with an interpreter in ANC, what was the primary mode of interpretation? (n=8)  Live professional via telephone / video  In-person professional interpreter | 8 (42%)  11 (58%)  5 (63%)  3 (38%) |
| *Needing an interpreter for Intrapartum Care (n=16):*  Interpreter present in intrapartum period?  Yes  No  For those with an interpreter in the intrapartum period, what was the primary mode of interpretation? (n=3)  In-Person professional interpreter  Was an interpreter present for consent for all intrapartum interventions? (n=3)  Yes  No | 3 (19%)  13 (81%)  3 (100%)  0 (0%)  3 (100%) |
| *Needing an interpreter for Postpartum Care (n=9):*  Interpreter present in postpartum period?  Yes  No | 0 (0%)  9 (100%) |
| * Percentages may not add to 100 given rounding.  ** Interpreter used at all antenatal visits and phone calls. | |

| **Appendix 4.3: Antenatal Care in Most Recent Pregnancy. (N=30 women).** | |
| --- | --- |
|  | **n (%)*** |
| First antenatal appointment 13 weeks gestation or more)  Yes  No | 19 (70%)**  8 (30%) |
| Continuity of carer (e.g. single designated midwife or obstetrician)  Yes  No  Not applicable  Not recorded | 4 (13%)  24 (80%)  1 (3%)  1 (3%) |
| Mental health assessed  Yes  No  Not recorded  If assessed, were formal validated mental health screening questionnaires used (e.g. Whooley Questions)? (n=18)  Yes  No | 18 (60%)  11 (37%)  1 (3%)  14 (78%)  4 (22%) |
| *Maternity Notes Including a Prompt to Ask About Complex Social Factors in Medical Records:*  Prompt to ask About Financial Need:  Yes  No  Prompt to ask About Housing Status:  Yes  No  Prompt to ask if Living in Asylum / Refugee Accommodation:  Yes  No  Prompt to ask About Criminal Justice Involvement:  Yes  No  Prompt to ask About Social Services Involvement  Yes  No  Prompt to ask About Interpersonal / domestic violence  Yes  No  Prompt to ask About Victim of trafficking / modern slavery  Yes  No | 3 (10%)  27 (90%)  13 (43%)  14 (47%)  5 (17%)  25 (83%)  3 (10%)  27 (90%)  20 (67%)  10 (33%)  11 (28%)  19 (63%)  0 (0%)  30 (100%) |
| Interpersonal / domestic violence documented  Yes  No  Not assessed | 3 (10%)  8 (27%)  19 (63%) |
| Birth plan documented (only relevant to women still receiving ANC at 34 weeks gestation or later) (n=21)  Yes  No | 2 (10%)  19 (91%) |
| * Percentages may not add to 100 given rounding.  ** Includes only those who were not pregnant on arrival to the UK (n=27) | |

| **Appendix 4.4: Intrapartum care in most recent pregnancy. (N=30 women).** | |
| --- | --- |
|  | **n (%)*** |
| Mode of birth  Caesarean section  Vaginal birth  Vaginal breech  Not applicable  Not recorded | 17 (57%)  6 (20%)  2 (7%)  3 (10%)  2 (7%) |
| Was labour induced?  Yes  No  Not recorded | 5 (17%)  20 (67%)  5 (17%) |
| * Percentages may not add to 100 given rounding. | |

| **Appendix 4.5: Postpartum care in most recent pregnancy. (N=8 women).** | |
| --- | --- |
|  | **n (%)*** |
| *Postpartum Mental Health Assessment:*  Mental health assessed  Yes  No  If assessed, were formal validated mental health screening questionnaires used (e.g. Whooley Questions)? (n=4)  Yes  No | 4 (50%)  4 (50%)  2 (50%)  2 (50%) |
| Continuity of midwifery care  Yes  No  Not applicable | 2 (25%)  5 (63%)  1 (13%) |
| * Percentages may not add to 100 given rounding. | |

| **Appendix 4.6: General overview of care received. (N=39 women)*.** | |
| --- | --- |
|  | **n (%)**** |
| NHS maternity service involved  Yes  No  If no, why not? (n=8)  Termination of pregnancy  Arrived in postpartum period  Private maternity care  Early pregnancy loss | 31 (80%)  8 (21%)  4 (50%)  2 (25%)  1 (13%)  1 (13%) |
| Local review of maternal death carried out  Yes  No  Not recorded  Not applicable | 23 (59%)  12 (31%)  3 (8%)  1 (3%) |
| Birthing partner present, where applicable (n=24)  Yes  No  Not recorded | 14 (58%)  3 (8%)  7 (29%) |
| Was the woman admitted to critical care?  Yes  No | 18 (60%)  12 (40%) |
| Was the infant stillborn?  Yes  No  Not recorded | 2 (7%)  23 (77%)  5 (17%) |
| Was the infant admitted to critical care? (n=23)  Yes  No | 12 (52%)  11 (37%) |
| * Unless not eligible for this assessment.  ** Percentages may not add to 100 given rounding. | |

**References**

1.	*Microsoft Excel*. 2018. <https://office.microsoft.com/excel>

2.	UK Government. 2021 Census:  List of ethnic groups. Accessed 27-11-23, <https://www.ethnicity-facts-figures.service.gov.uk/style-guide/ethnic-groups#2021-census>

3.	The World Bank. World Bank Country and Lending Groups. <https://datahelpdesk.worldbank.org/knowledgebase/articles/906519>

4.	The National Institute for Health and Care Excellence. Pregnancy and complex social factors: a model for service provision for pregnant women with complex social factors. In: NICE, editor. CG1102010.

5.	NHS Digital. 1.13 Antenatal assessments within 13 weeks - NHS Digital. @NHSDigital. Accessed 8-12-23, <https://digital.nhs.uk/data-and-information/publications/statistical/ccg-outcomes-indicator-set/june-2020/domain-1-preventing-people-from-dying-prematurely-ccg/1-13-antenatal-assessments-within-13-weeks>

6.	Sandall J, Soltani H, Gates S, Shennan A, Devane D. Midwife-led continuity models versus other models of care for childbearing women. *The Cochrane database of systematic reviews*. 04/28/2016 2016;4(4)doi:10.1002/14651858.CD004667.pub5

7.	National Institute for Health and Care Excellence. Overview | Antenatal care | Quality standards | NICE. NICE. Accessed 27-11-23, <https://www.nice.org.uk/guidance/qs22>

8.	National Institute for Health and Care Excellence. Antenatal and postnatal mental health: clinical management and service guidance. 2022;
